# Profilometric and scanning electron microscopy analysis comparing hydroxyapatite and zinc oxide nanoparticles for erosion resistance

**DOI:** 10.1186/s12903-025-06299-2

**Published:** 2025-06-21

**Authors:** Mayar H. Hassaan, Nagah A. Rashad, Afaf A. El Sawa, Lubna M. Eldesouky, Aya S. Sedik

**Affiliations:** 1 Department of Oral Biology, College of Dentistry, Arab Academy for Science, Technology & Maritime Transport, El Alamein, Egypt; 2 Department of Oral Biology, Faculty of Dentistry, Alexandria University, Alexandria, Egypt; 3 Department of Pharmaceutics, Faculty of Pharmacy, Alexandria University, Alexandria, Egypt

**Keywords:** Dentin hypersensitivity, Nanoparticles, Zinc oxide nanoparticles, Hydroxyapatite nanoparticles, Dentinal tubules

## Abstract

**Background:**

Dentin hypersensitivity (DH) is a prevalent dental issue characterized by sharp pain of short duration. Although the exact cause of DH remains debated, the hydrodynamic theory explains its mechanism. Furthermore, various methods, such as potassium-containing toothpaste, mouthwash, and chewing gum, have been attempted to address DH. However, recent research has explored the potential of nanoparticles (NPs) for DH treatment due to their biocompatibility and bioactive properties.

**Methods:**

Twenty-seven sound premolars extracted for orthodontic purposes were utilized in the present study to obtain 27 mid-coronal dentin discs. These discs were then etched with 37% orthophosphoric acid for 20 s to expose dentinal tubules (DTs) and simulate DH. Subsequently, dentin discs were rinsed with distilled water for 1 min. These dentin discs were randomly allocated into 3 groups: Group I (etched control), Group II (the etched dentin discs were treated with a carboxymethyl cellulose (CMC) dental hydrogel scaffold loaded with 20% HANPs), and Group III (the etched dentin discs were treated with a CMC dental hydrogel scaffold loaded with 20% ZnO NPs). After 7 days, Group II and III underwent erosive challenge to evaluate the protective effect of the nanoparticle treatments. Finally, the dentin discs were analyzed using profilometric analysis to measure surface roughness (SRa), scanning electron microscopy (SEM) to evaluate DT occlusion, computer-assisted SEM image analysis using Image J.

**Results:**

Among the groups, Group I exhibited the highest SRa following the DH simulation. Group II showed lower SRa compared to group III. SEM analysis indicated that Group III displayed more occluded DTs compared to Group II. Group I showed the most unoccluded DTs. The SEM analysis results were further quantitatively confirmed using Image J.

**Conclusions:**

The results indicated that both HANPs and ZnO NPs exhibited promising outcomes; however, ZnO NPs demonstrated superior effectiveness in resisting erosive wear compared to HANPs.

**Supplementary Information:**

The online version contains supplementary material available at 10.1186/s12903-025-06299-2.

## Highlights

The inclusion of ZnO NPs is anticipated to enhance clinical outcome in reducing dentine hypersensitivity by occluding dentinal tubules and resist erosive challenges.

All authors agree with the submission.

The work has not been published or submitted for publication elsewhere, either completely or in part, or in another form or language.

No conflict of interest with the authors in this work.

**Supplementary Information:**

The online version contains supplementary material available at 10.1186/s12903-025-06299-2.

## Background

The main structural component of a human tooth is dentin. It is composed of 67% inorganic and 33% organic materials [1]. It is a mineralized tissue mostly made up of DTs, which are in charge of hydration and tooth sensory responses [2]. Moreover, dentin acts as a passive barrier to protect the dental pulp [1].

Dentin hypersensitivity is described as a temporary, acute discomfort, accompanied by thermal, mechanical, chemical, and osmotic stimulation [3]. The cause of DH is still controversial; however, several hypotheses were suggested to explain DH as direct innervation theory, odontoblast transducer theory and hydrodynamic theory, which is the most widely accepted [4].

Furthermore, DH is often brought on by a combination of predisposing conditions as enamel loss as in case of attrition, abrasion, and erosion [5]. In addition, vigorous tooth brushing, acidic food, acids or medications may also lead to DH [6]. Therefore, all these etiological factors must be addressed before beginning DH therapy [7].

The prevalence of DH varies among different researches significantly, ranging from 1.3 to 92.1%, according to sociodemographic attributes, recruitment methodologies, and the quantity of study locations engaged [8]. It is more common on the buccal cervical zones of the permanent teeth and canines are most commonly affected [7].

Dentin hypersensitivity treatments are categorized by mechanism, including nerve desensitization, protein precipitation, tubule occlusion, adhesive sealers, laser treatments, and homeopathic options like propolis [9].

Moreover, treatment of DH is classified according to the mode of administration into: Home-based desensitization therapy and Office-based desensitization therapy [9].

Home-based desensitization therapy includes chewing gum, dentifrices, and mouthwashes. Regular assessment of the home-based treatment should be conducted every 3 to 4 weeks. However, if DH does not show any improvement to home-based therapy; then, it is advisable to initiate office-based therapy [7, 9].

Office-based desensitization therapy helps prevent DH from dental procedures, using agents like fluorides, varnishes, and lasers. However, achieving durable, effective protection against DH remains challenging [9–11].

Therefore, nanoparticles have been discovered to offer a promising treatment for DH [12]. NP possess small dimensions, increased surface area and high surface energy [13]. Nanoscale materials are increasingly favored for delivering antimicrobials and remineralizing agents into DTs due to their enhanced penetration, offering improved remineralization and reduced sensitivity [13]. The high surface energy and large surface area of NPs enhance their solubility, reactivity, and ability to deposit on uneven surfaces [14].

Several nanoparticles were used earlier to treat DH such as hydroxyapatite (HA) [15], doxycycline-doped polymeric NPs and calcium-doped polymeric NPs. Calcium-doped polymeric nanoparticles effectively block DTs by forming calcium-phosphate deposits but do not improve dentin’s mechanical properties or remodeling. Doxycycline serves as both an antibacterial and dentin matrix metalloproteinases (MMP) inhibitor, but leaves many DTs unblocked and does not lower hydraulic conductance [16]. In most cases, mineral precipitates are weakly deposited and do not adhere to the matrix [3].

Hydroxyapatite nanoparticles are utilized to treat DH [17] due to the fact that it is the main mineral in human teeth [18]. It shows several advantages such as bioactivity, biocompatibility [19], better wear performance and reduced cost, in comparison to materials typically utilized in oral healthcare [15]. However, it was discovered that the deposits created by HA paste were not resistant to acidic and mechanical stresses [20]. Additionally, a previous study on the HA’s ability to promote remineralization revealed that its effectiveness in the overall remineralization process was comparatively low [21].

In order to overcome the limitations of the current agents, it is important to create an occlusion agent that encourages the growth of a deep and compact mineralized occlusion layer of DTs [22], be painless, easy to use, long-lasting, and not damage the teeth or gingiva [15].

Zinc oxide nanoparticles are a type of metal oxide NPs that offer novel possibilities in the field of biomedical applications, including diagnostics and therapeutic interventions [23]. ZnO NPs has generated a lot of attention due to its affordability, antibacterial properties, and general safety [24].

Furthermore, zinc oxide nanoparticles are widely used in dentistry to boost antibacterial effects in restoratives, act as desensitizers in toothpastes, fight oral pathogens, aid in remineralizing dentinal lesions, and improve drug delivery stability [23]. Moreover, the zinc (Zn) in the metal oxide can assist prevent collagen breakdown and enhance dentin remineralization [25], as well as occlusion of DTs [16].

Hence, the objective of the current study was to evaluate the effectiveness of ZnO NPs in comparison to HANPs in occluding DTs and their ability to withstand erosive wear.

The null hypothesis proposed in this study asserts that no significant differences exist among the various experimental groups.

## Materials and methods

Twenty-seven sound premolars extracted for orthodontic purpose were used in this study to obtain twenty-seven dentin discs. Teeth were obtained from the Oral and Maxillofacial Surgery Department, Faculty of Dentistry, Alexandria University. The study was conducted after receiving the approval of the Ethical Committee at Faculty of Dentistry, Alexandria University. (IRB No. 001056–IORG 0008839) (0538 − 11/2022)

The randomization scheme used to allocate the 27 dentin discs was generated by using the website (http://www.Randomization.com), dividing them into 3 equal groups, 9 dentin discs per group.

- **Group I**: (*n* = 9) Etched control group.
- **Group II**: (*n* = 9) Hydroxyapatite nanoparticles group.
- **Group III**: (*n* = 9) Zinc oxide nanoparticles group.

### Preparation of artificial saliva ( [26])

Artificial saliva was prepared using Macknight-Hane and Whitford’s (1992) formula, which included methyl-p-hydroxybenzoate, sodium carboxymethyl cellulose, potassium chloride, magnesium chloride, calcium chloride, dipotassium hydrogen phosphate, and potassium dihydrogen phosphate.

### Orthophosphoric acid Preparation (37%) [27]

To prepare a 25 mL solution of 37% orthophosphoric acid, 9.25 mL of orthophosphoric acid was added to a flask, and water was gradually added to reach 25 mL, followed by homogenization using a Sonicator.

### Preparation of carboxymethyl cellulose dental hydrogel scaffold ( [15])

Carboxymethyl cellulose (CMC) dental hydrogel scaffold was prepared by using 0.8 g of CMC, 4 g of glycerol and 11.2 g of H2O added in a beaker. The mixture was then thoroughly blended using a magnetic stirrer.

### Preparation of carboxymethyl cellulose dental hydrogel scaffold loaded with 20% hydroxyapatite nanoparticles ( [15])

To create a homogeneous mixture, 4 g of HANPs (purchased from Nanotech, Cairo, Egypt) was added to the beaker that contained CMC dental hydrogel scaffold and was continuously mixed for 1 h using a magnetic stirrer, transforming into a paste.

### Preparation of carboxymethyl cellulose dental hydrogel scaffold loaded with 20% zinc oxide nanoparticles ( [15])

To prepare CMC dental hydrogel scaffold loaded with 20% ZnO NPs, 4 g of ZnO NPs (purchased from Nanotech, Cairo, Egypt) was added to the beaker that contained the CMC dental hydrogel scaffold and was continuously mixed for 1 h using a magnetic stirrer until a homogeneous consistency was achieved, transforming into paste.

### Dentin disc Preparation

The present research was applied on 27 extracted sound premolars that were collected from Oral and Maxillofacial Surgery Clinics for orthodontic reasons to obtain 27 dentin discs [4]. Teeth were stored in 10% Formalin [28]. The coronal portion of the teeth was exposed by mounting extracted premolars to acrylic molds. A Microtome (Micracut 150, Metkon^®^ Metallography, Bursa, Turkey) was used to remove 2.5 mm of occlusal enamel, down the cusp tip. Dentin discs of 2.0 mm (± 0.2 mm) in size; were prepared by cutting each tooth horizontally (mesio-distally) [4], from mid-coronal part [29], over cemento-enamel junction (CEJ) [27]. Dentin discs were ultrasonically cleaned and rinsed with deionized water [15].

### Etching procedure

Dentin discs were etched with 37% orthophosphoric acid for 20 s to open the DTs, to create a DH model [4] and to remove the smear layer [30]. Dentin discs were then washed with distilled water for 1 min [4]. Then they were ultrasonically cleaned for 15 min. Afterwards, dentin discs were kept in artificial saliva to simulate the oral conditions [31].

### Simulated tooth brushing protocol for dentin discs ([15])

Etched dentin discs were marked on the non-brushing side with a corrector to ensure consistent positioning during each brushing cycle, mounted on a rubber base, and brushed with CMC dental hydrogel scaffolds loaded with HANPs (Group II) and ZnO NPs (Group III), using artificial saliva to simulate oral conditions. The volume of the pea-sized amount of the CMC dental hydrogel scaffold loaded with HANPs or ZnO NPs was carefully controlled and measured using a calibrated electronic balance during the preparation phase to ensure consistent application across all specimens. Brushing cycles were performed for 2 min, 3 times daily for 7 days, using a custom-made brushing simulator, with the brush size covering the entire surface of the disc. After each cycle, the discs were washed with deionized water and returned to containers with artificial saliva.

### Citric acid Preparation (0.3%) [32]

To prepare 0.3% citric acid, 75 mg of citric acid powder was dissolved in water to a 25 mL volume, and the pH was measured as 2.60 using a METTLER TOLEDO pH meter.

### Erosive challenge

Dentin discs were immersed in 10 mL of 0.3% citric acid solution (pH = 2.6) for 2 min [32], rinsed with deionized water [15], and gently dried with absorbent paper. Afterwards, specimens were immersed in artificial saliva to simulate oral conditions. This procedure was repeated 4 times a day, for 5 days without stirring at room temperature [32].

### Methods of specimens’ examination

#### Profilometric analysis of dentin discs using 3d laser scanning microscope for surface roughness evaluation ( [32])

Topographical data was extracted using a 3D laser scanning microscope (Keyence VK-X100, KeyenceGmbH, Neu-Isenbuerg, Germany) at Egypt- Japan University of Science and Technology, New Borg El-Arab City, Egypt. It was used to measure the SRa of dentin discs, with three areas on each specimen analyzed at 20X magnification; roughness values were averaged, and visual surface images were captured for qualitative assessment, using a color scale to differentiate pits and peaks.

#### Characterization of carboxymethyl cellulose dental hydrogel scaffold by scanning electron microscope ( [33])

##### Scanning electron microscopic procedure

Characterization of the CMC dental hydrogel scaffold, loaded with HANPs and ZnO NPs, was performed using JEOL SEM (JSM-IT200 Series) at the Faculty of Science, Alexandria University, Alexandria, Egypt. It was used to evaluate the size and shape of the nanoparticles, with the scaffolds coated with a gold layer and mounted for examination.

#### Scanning electron microscopic assessment of dentin discs for surface morphological evaluation ( [15, 34])

##### Preparation for scanning electron microscopy

Dentin discs were washed with distilled water, dried at room temperature, freeze-dried, and mounted on an aluminum stub using double-sided carbon tape. Using a sputter coater, mounted dentin discs were given ultra-thin coating of gold to prevent electrostatic charge accumulation [35]. Dentin discs were examined after the completion of the entire study, meaning that for Group I (the etched control group), the measurements were taken after etching, while for Groups II and III (treated with a CMC dental hydrogel scaffold loaded with nanoparticles) using JEOL SEM to qualitatively assess the degree of DT occlusion, ensuring the coating was thin enough to preserve surface layer resolution.

#### Computer-assisted scanning electron microscopic analysis of dentin discs ( [36, 37])

To standardize the quantitative evaluation of the degree of DT occlusion, image analysis was performed by a computer software platform (Image J 1.53k, NIH, Bethesda, MD, USA) on a five-grade scale, according to the tubule occlusion classification scoring system used previously by Vasu Midha and Hemalatha Doppalapudi, since this scoring system is employed in the most recent research studies, it is considered the prevailing method.

**Table:** 

Score	Classification	Description
1	Occluded	Complete tubular closure (100% of the tubules are closed)
2	Mostly Occluded	Significant closure (50% to less than 100% of tubules closed)
3	Partially Occluded	Moderate closure (25% to less than 50% of tubules closed)
4	Mostly Unoccluded	Minimal closure (less than 25% of tubules closed)
5	Unoccluded	No closure (0% of tubules closed; fully open)

#### Statistical analysis

The Profilometric and Image J analysis results were collected and analyzed using One Way ANOVA. All tests were two tailed and level of significance was set at *p* value < 0.05. Data were analyzed using IBM SPSS version 28, Armonk, NY, USA.

## Results

### Characterization of nanoparticles

#### Characterization of carboxymethyl cellulose dental hydrogel scaffold loaded with hydroxyapatite nanoparticles

**Fig. 1:**
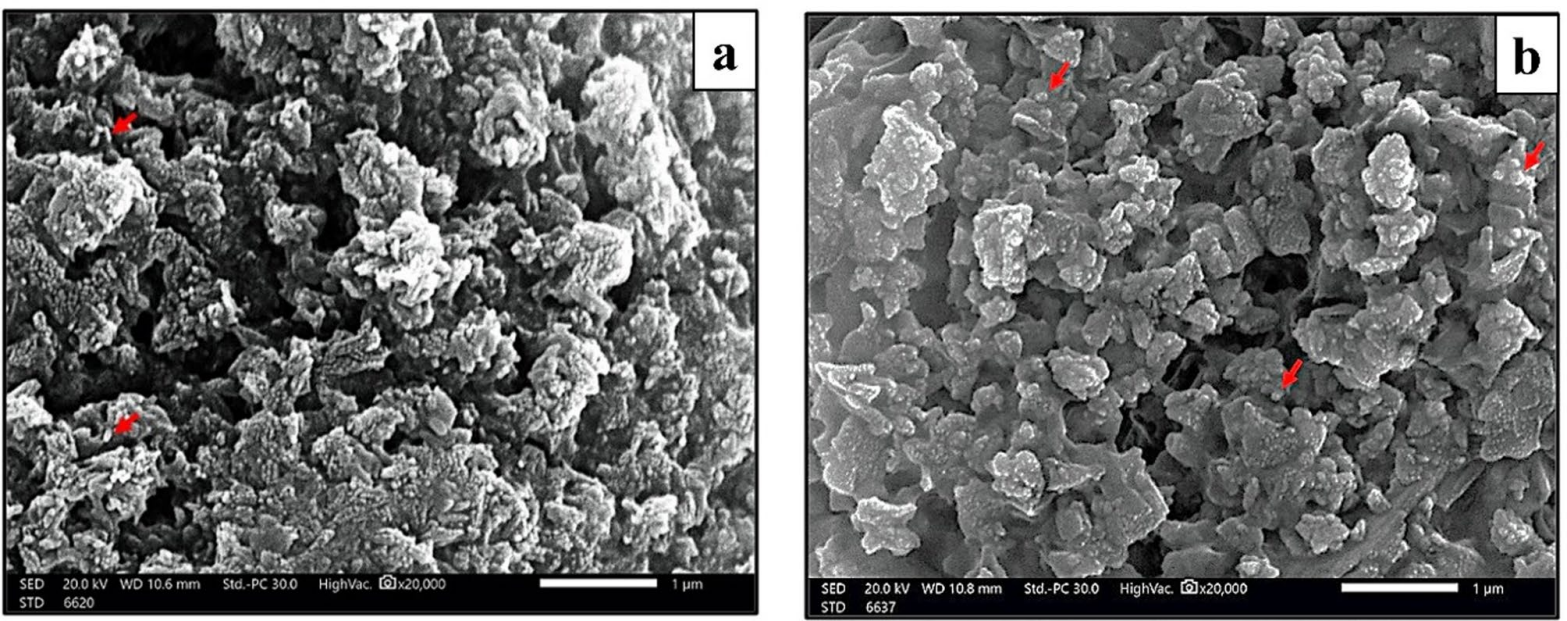
Characterization of nanoparticles by SEM. SEM image shows rod-shaped HANPs (arrows) homogeneously distributed and cross-linked within the CMC dental hydrogel scaffold (Scale bar; 1 μm) (**a**). SEM image shows spherical ZnO nanoparticles (arrows) uniformly distributed and cross-linked within the CMC dental hydrogel scaffold (Scale bar; 1 μm) (**b**)

Scanning electron micrographs of the CMC dental hydrogel scaffold loaded with HANPs, confirming the HANPs size range 25 ± 5 nm and providing visual evidence of the uniform dispersion of the rod-shaped HANPs throughout the scaffold, ensuring a consistent distribution. (Fig. 1a)

#### Characterization of carboxymethyl cellulose dental hydrogel scaffold loaded with zinc oxide nanoparticles

Scanning electron micrographs of the CMC dental hydrogel scaffold loaded with ZnO NPs, confirm the size ranging 30 ± 10 nm and homogenous distribution of the spherical shaped ZnO NPs among the scaffold. (Fig. 1b)

### Profilometric analysis results

A statistically significant difference in SRa was observed between the study groups, with a *p*-value of ≤ 0.05.

**Fig. 2:**
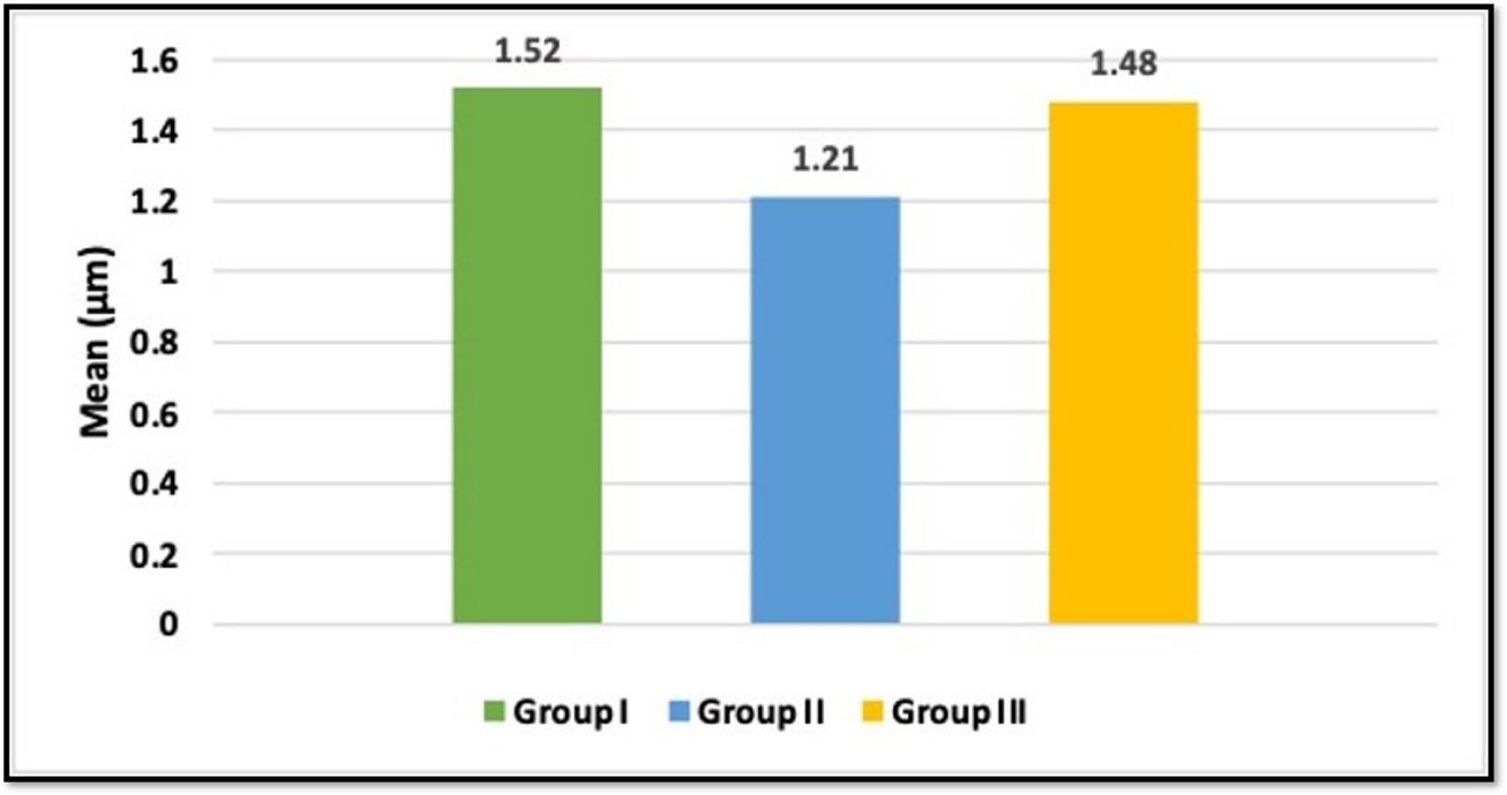
Comparison of surface roughness among the study groups, represented in the bar chart

Group I, the etched control group, showed the highest SRa, with a mean value of 1.52 ± 0.33 μm. Group II, displayed a mean value of 1.17 ± 0.11 μm, while Group III, displayed a mean value of 1.48 ± 0.40 μm, which were summarized in a bar graph. (Fig. 2)

**Fig. 3:**
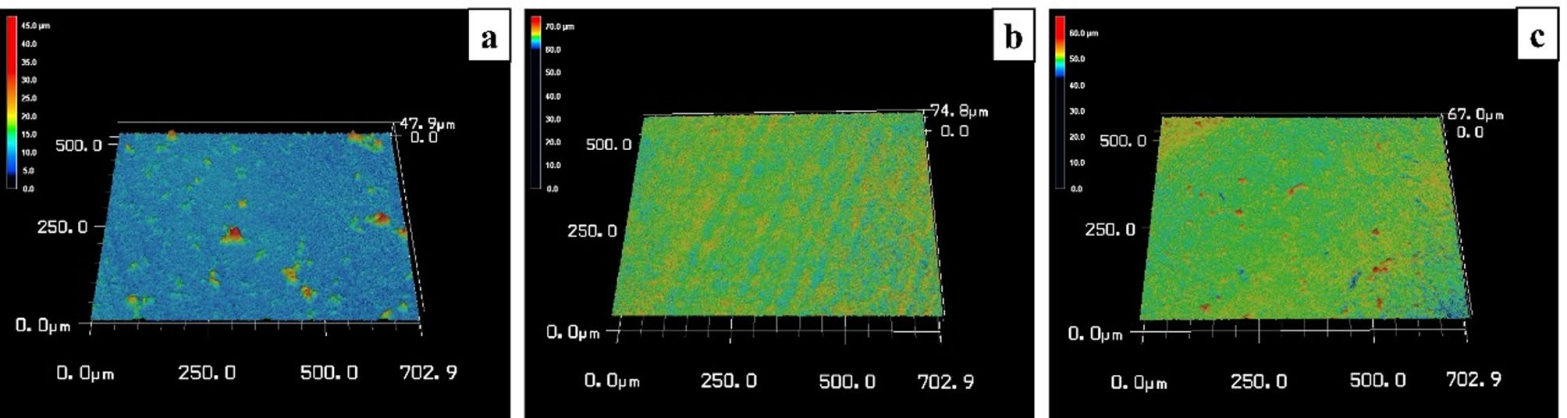
Profilometric analysis. Profilometric image of Group I shows the highest SRa values, with blue indicating pits and red indicating peaks on the dentin surface (**a**). Profilometric image of Group II shows a significant reduction in SRa, with the lowest roughness among all groups and minimal red peaks (**b**). Profilometric image of Group III shows a decreased SRa compared to Group I, with more red peaks than Group II (**c**)

Profilometric images showing SRa across the different groups revealed the following: In Group I, the image exhibited the highest SRa, with blue highlighting pits and red representing peaks (Fig. 3a). Group II demonstrated a reduction in SRa of dentin discs following brushing with HANPs (Fig. 3b). Group III also displayed a decrease in SRa of dentin discs after brushing with ZnO NPs (Fig. 3c).

### Scanning electron micrograph results

SEM images of dentin discs from all study groups were selected and captured at a magnification of 5000× to evaluate their surface features and the effectiveness of the NPs in sealing the DTs.

#### Group I

**Fig. 4:**
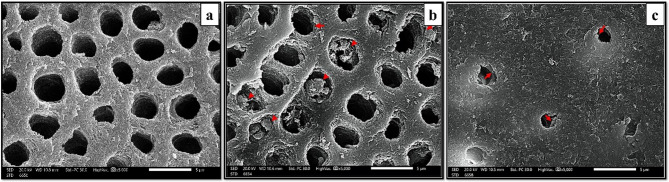
Scanning electron micrograph. SEM image of Group I showing demineralized dentin surface with patent DTs and collapsed collagen network (x5000) (**a**). SEM image of Group II shows partial occlusion of dentinal tubules with HANP precipitates (arrowheads), alongside areas of HANP dislodgement causing tubule damage (**b**). SEM image of Group III shows ZnO NP precipitates inside dentinal tubules (arrows), mostly occluding them with some partially closed (**c**)

Dentin discs exhibited patent DTs with some exposure to the collagen fibers, confirming the demineralization and replication of DH model. (Fig. 4a)

#### Group II

The dentin discs showed partial occlusion of DTs due to the presence of HANP precipitates displaying resilience against the acidic attack, managing to retain the HANP plugs. (Fig. 4b)

#### Group III

The dentin discs demonstrate dissolved ZnO NPs inside the DTs. Consequently, the majority of the DTs became occluded by these precipitated ZnO NPs, while only a few remained partially closed or reduction in the diameter of DTs. (Fig. 4c)

### Computer-assisted scanning electron microscopic results (statistical analysis)

Different quantitative analysis of the DTs occlusion was found in the various groups, according to Image J.

According to the percentages of the degree of DTs occlusion, which were summarized in the bar graph, the study groups were compared [38].

**Fig. 5:**
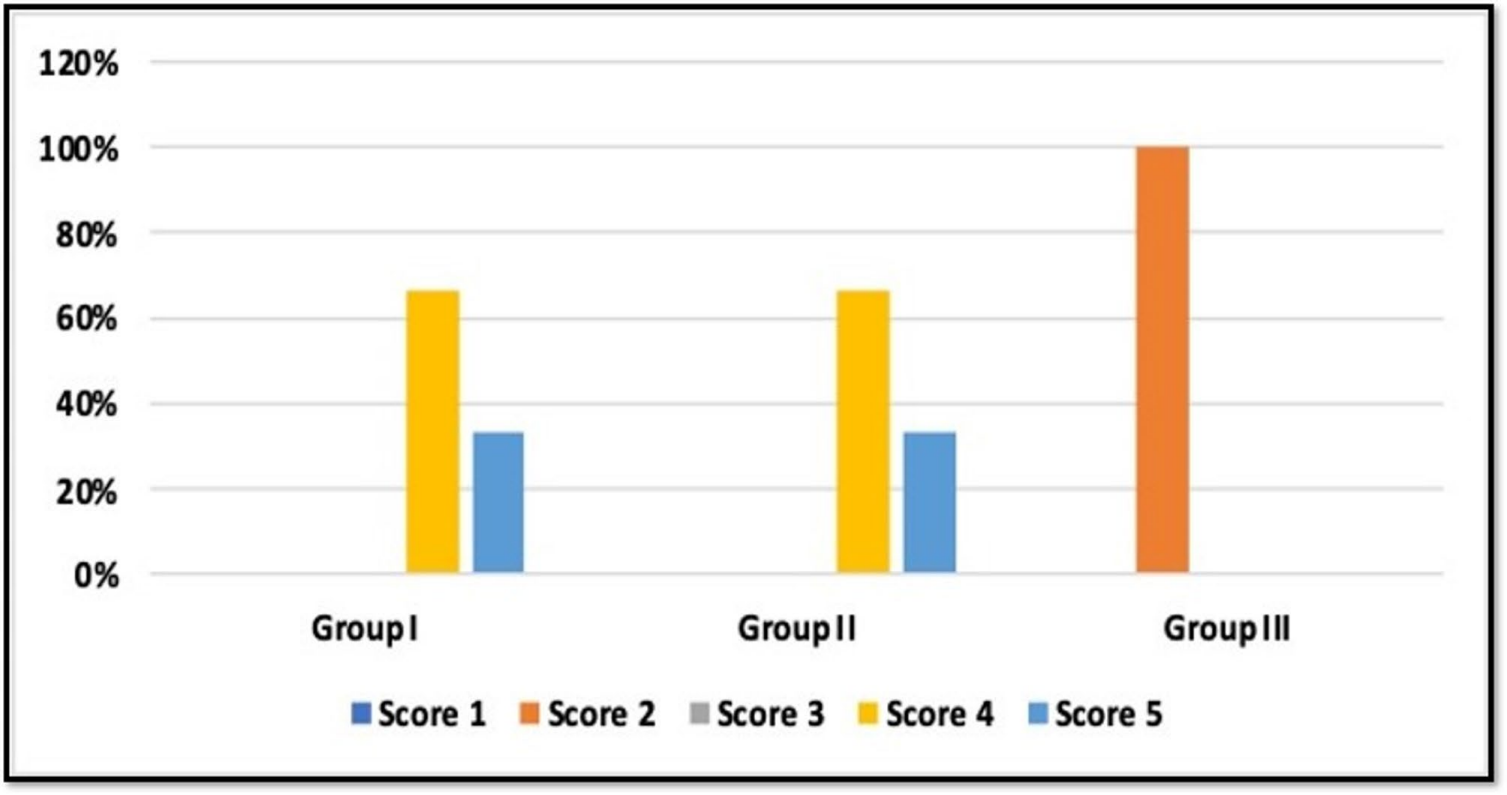
Bar chart presenting the quantitative comparison of dentinal tubule occlusion among the study groups

Group I (etched control) showed 66.7% of the dentin discs scored 4, while 33.3% scored 5. Group II (HANPs) showed 66.7% of dentin discs scoring 2, while 33.3% scoring 3. Group III (ZnO NPs) revealed that 88.9% of dentin discs scored 1, while only 11.1% scored 2. There was statistically significant difference between the study groups with *p* value ≤ 0.05. (Fig. 5).

## Discussion

Dentin hypersensitivity is a sharp, short-lasting pain from exposed dentin triggered by thermal, tactile, osmotic, or chemical stimuli, often caused by gingival recession, abrasive brushing, or acidic exposure near the CEJ [39]. Gastric reflux and psychological conditions like bulimia nervosa can lead to dental erosion, abrasion, and gingival recession by exposing teeth to stomach acids, making it crucial to identify and address these factors for effective DH management [40].

Previous studies have explored the use of ZnO NPs as desensitizing agents in toothpaste formulations. For instance, Toledano-Osorio et al. (2021) [41] demonstrated that toothpastes containing Zn-doped NPs effectively occluded DTs and enhanced the mechanical properties of dental structures in vitro. Similarly, HANPs have been investigated for their potential in managing DH. A study by Onwubu et al. (2019) [42] showed that HANPs synthesized from eggshell waste effectively occluded DTs in vitro.

However, despite these promising findings, the literature lacks sufficient evidence regarding the capability of both ZnO NPs and HANPs to resist erosive challenges. Most existing studies focus on their immediate desensitizing effects, with limited research assessing their long-term durability under erosive conditions. Moreover, a direct comparison between the two materials under identical testing protocols is notably absent, making it difficult to determine which NP type performs better in terms of both occlusion and resistance to acidic conditions.

The clinical significance of comparing HANPs and ZnO NPs in DH treatment lies in their potential to provide long-lasting relief and improve patients’ oral health-related quality of life. While both NPs have demonstrated efficacy in occluding DTs and reducing sensitivity, there is a lack of comprehensive studies directly comparing their resistance to erosive challenges. Understanding their comparative performance under such conditions is crucial for developing more effective desensitizing agents. Further research in this area could lead to optimized formulations that offer enhanced durability and sustained relief for individuals suffering from DH.

Therefore, our study compared the efficacy of HANPs and ZnO NPs in resisting erosive challenges, evaluated through SRa using profilometric analysis, SEM analysis, and ImageJ software.

The study involved 27 dentin discs, treated with 37% orthophosphoric acid for 20 s to expose DTs, remove the smear layer, and replicate the conditions of DH [31]. The dentin discs were randomly divided into two groups, with Group I serving as the etched control, while Groups II and III underwent an erosive challenge after 7 days.

The above mentioned aligns with the approach used by Khan et al. (2020) [4], who employed the same technique to simulate DH for the purpose of comparing the occlusion effectiveness of 2 experimental dentifrices. This is also consistent with Saikaew et al. (2022) [30], who demonstrated that in clinical practice, the commonly used and optimal concentration of phosphoric acid typically falls within the range of 30–40% for effectively removing the smear layer.

Alongside, the present study was conducted over 7 days, this is in accordance with Sadiasa et al. (2013) [15], who concluded that this time frame was considered suitable and affords a thorough assessment of CMC hydrogel’s potential as a dental gel for delivering HA into DTs, with the aim of alleviating or eradicating DH.

In addition, to replicate oral conditions, the dentin discs were placed in artificial saliva during the intervals between brushing cycles. This methodology aligns with the approach taken by Ashraf and Aidaros (2021) [34], who examined the effectiveness and durability of nano seashell, sodium fluoride and commercially available tooth paste in addressing DH. The procedure involved the application of paste onto the sample surface for a duration of 2 min, using a micro brush, then rinsing with deionized water for 30 s. Afterward, the samples were placed in a container filled with artificial saliva at 37 °C. This cycle was repeated every 12 h for a period of 14 days, and the artificial saliva was replaced every 24 h. They concluded that artificial saliva simulates conditions found within the oral cavity.

Besides, a brushing simulator was used to meticulously standardize the variables of applied force, duration, and the number of brushing cycles across all groups. This approach was in accordance with Farooq et al. (2021) [43], who assessed the remineralization effectiveness of a newly developed fluoridated bioactive glass toothpaste in comparison to a conventional fluoride toothpaste and implemented a simulated brushing protocol utilizing a mechanical brushing machine.

Additionally, to evaluate the resistance of the DTs to occlusion, dentin discs were exposed to acidic challenge [44]. Therefore, a 5-day erosive challenge for Groups II and III involved four 2-minute cycles per day, with dentin discs immersed in a 0.3% citric acid solution at pH 2.6. This is in agreement with Parkinson et al. (2011) [45], who studied the impact of 3 dietary acids and found that citric acid, among phosphoric, citric, and oxalic acids, had the most significant demineralizing effect on dentin, given that its critical pH level is 6.5.

This value is significant as it marks the pH level where saliva loses its saturation with calcium and phosphorus, potentially leading to tooth dissolution [46]. Moreover, citric acid is widely utilized in vitro to simulate an erosive challenge on enamel and dentin [47, 48], since it is a common component in many soft drinks and fruit juices consumed daily [49], with a pH range of 2.0 to 3.0, these acids can cause etching on exposed dentin surfaces [50].

This procedure was adapted from Scaramucci et al. (2013) [51], the study aimed to replicate the conditions of individuals with DH, where frequent consumption of acidic beverages and toothbrushing can open and widen DTs, potentially exacerbating DH and reducing treatment efficacy. In addition, Garofalo et al. (2019) [32], has indicated that the oral cavity’s pH typically remains low for about 2 min, and extending this duration may unrealistically alter the eroded surface.

SEM micrographs confirmed the size of HANPs was 25 ± 5 nm and showed their even dispersion within the CMC dental hydrogel scaffold. These results align with the research conducted by Balhuc et al. (2021) [52], who explored the role of HANPs in dentistry and confirmed their rod-shaped characterization through TEM and SEM, showing a size range of 20 to 80 nm.

SEM images confirmed that the ZnO NPs in the CMC dental hydrogel scaffold was 30 ± 10 nm in size and distributed homogeneously throughout the scaffold. This finding aligns with Yadollahi et al. (2015) [53], who confirmed the presence of distinct spherical particles in the nanocomposite hydrogel containing ZnO NPs.

Since the profilometric analysis for measuring the SRa serves as the most frequently employed parameter for providing a quantitative description [54–56], it is the preferred choice for the present study. The study found that Group I had the highest SRa, followed by Group III, while Group II exhibited the lowest SRa.

The profilometric analysis results for Group I, displayed the highest degree of SRa among all the study groups. This is in accordance with Farooq et al. (2021) [43], who concluded that citric acid-induced demineralization increased SRa, confirming that demineralization raises SRa levels.

In contrast, the profilometric analysis of Group III showed a decrease in SRa compared to Group I, but a higher SRa than Group II, with increased surface irregularities and roughness.

Correspondingly, the dissolution of the thick ZnO NP layer likely contributed to the significant variation in profilometric measurements observed in Group III.

This is in accordance with Garofalo et al. (2019) [32], who evaluated the impact of four in-office desensitizing products which revealed that some specimens experienced complete detachment of the material, causing significant variation in profilometric readings.

This effect was attributed to the erosive challenge, which had a detrimental impact on the smoothness of the dentin, leading to a rougher texture. This is consistent with Li et al. (2020) [57], who found that prolonged immersion in commercially available soft drinks caused a significant increase in the SRa of human teeth. Besides, this is in accordance with Farooq et al. (2021) [43], demonstrated that citric acid exposure caused dental blocks to become rougher due to demineralization, highlighting the impact of this process on surface texture in dental applications.

In addition to this, the increase in SRa observed could be attributed to the potential abrasiveness and chemical reactivity of ZnO NPs. Alongside, the findings of the present study align with Casamassa E et al. (2020) [58], the study found that ZnO powders, particularly spherical particles, increased SRa due to their abrasive nature, causing more significant surface damage and preventing the formation of a uniform layer at the interface.

After erosive challenges, Group III’s SRa decreased to 1.48, compared to Group I’s 1.52, despite Group III undergoing both initial etching and five days of exposure to 0.3% citric acid, demonstrating the superior resistance of ZnO NPs to erosive challenges.

Also, the results of the present study was confirmed by Farooq et al. (2021) [43], who reported consistent findings, indicating that the SRa increases following demineralization.

Profilometric analysis of Group II showed the lowest SRa, with the erosive challenge reducing irregularities and roughness, resulting in a smoother texture.

Group II dentin discs showed lower SRa than Group III, likely due to hydroxyapatite’s chemical stability in mildly acidic conditions, reducing the impact of citric acid [59], on HA might be generally less pronounced compared to its reactivity with ZnO, which might be more susceptible to acid-induced reactions. Correspondingly, this aligns with the findings of Skwarek et al. (2014) [60], their research found that citric acid ions selectively adsorb at the HA interface, leading to partial dissolution of the apatite structure.

Additionally, ZnO NPs show higher solubility in acidic solutions than HANPs, leading to greater dissolution and degradation when exposed to citric acid. The findings from Group III affirmed that Zn²⁺ ions became more prevalent and easily accessible in acidic settings. The availability of Zn ions in acidic environments impacts biological processes and the effectiveness of Zn-based materials [61].

The SRa findings of the present study were validated by the outcomes from studies conducted by Selivany BJ and Al-Hano F (2015) [62], who conducted a study and found that toothpaste with HANPs reduced SRa more effectively than NovaMin and Kin Sense fluoride after laser bleaching with 35% hydrogen peroxide.

The morphological evaluation of dentin discs was analyzed using SEM, dentin discs from each study group were selected to evaluate their surface topography and the efficiency of the NPs in sealing the DTs.

Scanning electron micrograph analysis of Group I showed the most exposed DTs, dentin degradation, and collapsed collagen, confirming demineralization and successful replication of the DH model.

The present research also confirmed the successful elimination of the smear layer, which is in line with the results of Kripal et al. (2019) [63], who explored the ability of propolis varnish to occlude DTs on dentin discs treated with 37% orthophosphoric acid, which effectively removed the smear layer and prepared the DTs for accurate assessment of occlusion.

Quantitative analysis was conducted to further support our SEM observations. To address the constraints encountered in the study by Khan et al. (2020) [4], which stemmed from the manual enumeration of obstructed DTs, a method susceptible to potential inaccuracies owing to human error. Computer-assisted SEM analysis was carried out using Image J software.

Quantitative SEM analysis for Group I showed 66.7% of dentin discs scored 4, indicating less than 25% of tubules were closed, while 33.3% scored 5, confirming a significant portion of DTs remained open, consistent with DH.

This is corroborated by Gergely et al. (2010) [64], who provided additional evidence that areas of DH exhibit less than 25% closure of DTs when examined through SEM.

SEM analysis of Group II showed partial resilience against acidity and low retention of HANP plugs, with HANPs less effective in sustaining DT occlusion compared to ZnO NPs, likely due to their smaller particle size and inability to form stable precipitates.

The results are consistent with Huang et al. (2011) [65], who found that HANPs do not effectively promote remineralization of subsurface lesions under neutral pH conditions, and high pH levels may lead to less stable surface layers.

Previous studies have demonstrated the superior DT occlusion potential of HANPs following simulated application. For example, Kunam et al. (2016) [66] evaluated the effectiveness of HANPs, in occluding DTs. The study used a 7 day treatment regimen and assessed the occlusion using SEM. The results showed that HANPs achieved tubule occlusion.

Another study by Onwubu et al. (2019) [42] demonstrated the effectiveness of HANPs in occluding dentinal tubules. In this in vitro study, HANPs synthesized from eggshell waste was applied to dentin specimens. Scanning electron microscopy showed that the treated samples had a significantly higher tubule occlusion compared to control groups. These results confirmed the potential of HANPs for managing DH.

Building on these findings, our study aimed to further investigate the resistance of HANP based scaffolds to erosive challenges following simulated tooth brushing. While the occlusive capacity of HANPs has been established in previous literature, our study complements this by focusing on their durability under more aggressive conditions.

The results of our current investigation find support with Ashraf et al. (2021) [34], who showed that dentin discs treated with a nano sea shell paste generating HA exhibited better DT occlusion after an acidic challenge than the etched control group, although occlusion was still lower than in the group not exposed to the acid.

Quantitative SEM analysis of Group II showed 66.7% of dentin discs scored 4, indicating less than 25% DT closure, while 33.3% scored 5, showing no tubular closure.

SEM analysis of Group III showed significant dissolution of ZnO NPs, leading to their precipitation within DTs and occluding most tubules, with fewer unoccluded DTs than in Group I and Group II, demonstrating ZnO NPs’ resistance to erosive challenges.

The larger particle size of ZnO NPs enables faster precipitate formation, suggesting their potential to effectively address DH, particularly under erosive conditions like citric acid exposure.

This is in accordance with Khan et al. (2020) [27], who conducted an in vitro study and found that dentifrices containing ZnO NPs demonstrated significant DT occlusion, even after citric acid exposure, compared to other experimental and commercially available dentifrices.

Previous studies, such as the one conducted by Khan et al. (2020) [4], utilized simulated tooth brushing over a period of 7 days to evaluate the DT occlusion potential of ZnO NPs. Their findings demonstrated that ZnO NPs exhibited a superior occlusive effect, even after an acid challenge, confirming their efficacy in managing DH. Specifically, SEM revealed that ZnO NP containing dentifrices achieved significant and consistent tubule occlusion compared to commercial controls.

Moreover, previous studies have demonstrated the efficacy of ZnO NPs in occluding DTs following simulated tooth brushing. For instance, Toledano-Osorio et al. (2021) [41] evaluated the effectiveness of dentifrices containing ZnO NPs in occluding DTs. Their findings revealed that ZnO NP containing dentifrices achieved significant tubule occlusion, even after exposure to an acid challenge, confirming their potential in managing DH.​.

The aim of our study was not to repeat prior evaluations of the occlusive potential of ZnO nanoparticles, which have already been well-documented, but to extend this research by assessing their resistance to erosive challenges following simulated tooth brushing. Since the occlusive ability of ZnO NPs is already established, evaluating their durability under more aggressive, real-world conditions is essential for determining their long-term clinical relevance and applicability.

In addition, the findings of the present study aligns with the findings of Toledano-Osorio et al. (2021) [67], who found that only dentifrices containing Zn-doped polymeric NPs effectively occluded DTs and resisted citric acid, while about 30% of tubules in commercially available toothpaste-treated specimens reopened.

Quantitative SEM analysis of Group III showed 100% of dentin discs scored 2, with 50% to nearly 100% of DTs closed, and reduced diameters of open tubules compared to Group I.

These results highlight that Group III achieved better DT closure and greater resistance to erosive wear than Group II, confirming the effectiveness of ZnO NPs in sealing DTs and enhancing resistance.

This is in accordance with Khan et al. (2020) [27], who conducted an in vitro study and found that dentifrices containing ZnO NPs effectively occluded DTs, with more than 50% but less than 100% of tubules remaining sealed after citric acid exposure.

The research found that HANPs were washed away and less effective than ZnO NPs in withstanding the erosive challenge.

In conclusion, the comprehensive analysis of profilometric data, scanning electron micrographs and computer-assisted SEM conducted in this research strongly supports the promising prospects of utilizing HANPs and ZnO NPs. However, ZnO NPs offered greater protection to the dental pulp from DT exposure compared to HANPs. Nevertheless, HANPs exhibited superior surface quality due to lower SRa. Furthermore, it suggests that this approach could potentially alleviate or completely eliminate DH while demonstrating remarkable resilience to the consumption of acidic substances in daily life.

### Limitations

The limitations of this study include the short treatment duration of only 7 days, which may not reflect long-term effectiveness, and the in vitro design, which may not fully replicate the complexities of the oral environment. The study also lacked clinical validation, as it was based on extracted human premolars.

Future studies should focus on investigating the long-term effects of ZnO NPs and HANPs on DTs by extending the treatment duration and exploring different NP concentrations. Comparative studies between various types of NPs, as well as molecular-level investigations of their interactions with dentin, would enhance our understanding of their mechanisms of action. Additionally, exploring the potential synergistic effects of combining NPs with other desensitizing agents could offer more effective treatment solutions for DH. Moreover, further studies can compare between commercially available fluoride-based products and ZnO NPs.

## Conclusions

The HANPs group showed improved surface quality with a smoother dentin surface, while the ZnO NPs group demonstrated better resistance to erosive wear, with the goal of finding an efficient, minimally invasive, and affordable solution for preserving dentin structure.

## Electronic supplementary material

Below is the link to the electronic supplementary material.

Supplementary Material 1

Supplementary Material 2

Supplementary Material 3

Supplementary Material 4

Supplementary Material 5

Supplementary Material 6

## Data Availability

All data sets and materials employed or examined in the present study are comprehensively documented within this published article.

## Abbreviations

DH: Dentin Hypersensitivity
NPs: Nanoparticles
DTs: Dentinal Tubules
HANPs: Hydroxyapatite Nanoparticles
ZnO NPs: Zinc Oxide Nanoparticles
SRa: Average Surface Roughness
SEM: Scanning Electron Microscopy
HA: Hydroxyapatite
MMP: Matrix metalloproteinase
Zn: Zinc
TEM: Transmission Electron Microscopy
CMC: Carboxymethyl Cellulose
CEJ: Cemento- Enamel Junction
