# Supplementary figures and images for: Profilometric and scanning electron microscopy analysis comparing hydroxyapatite and zinc oxide nanoparticles for erosion resistance
Source: BMC Oral Health. 2025 Jun 21;25:949. doi: 10.1186/s12903-025-06299-2 (PMC12182678; doi:10.1186/s12903-025-06299-2)

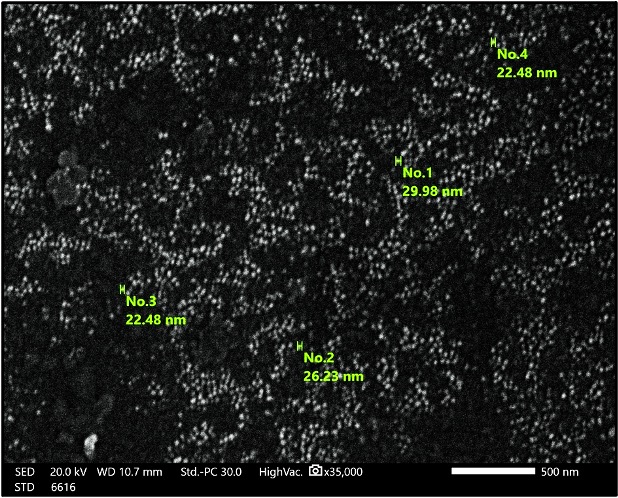

Supplement: Supplementary file 1 — Supplementary Material 1 [file 12903_2025_6299_MOESM1_ESM.jpg]

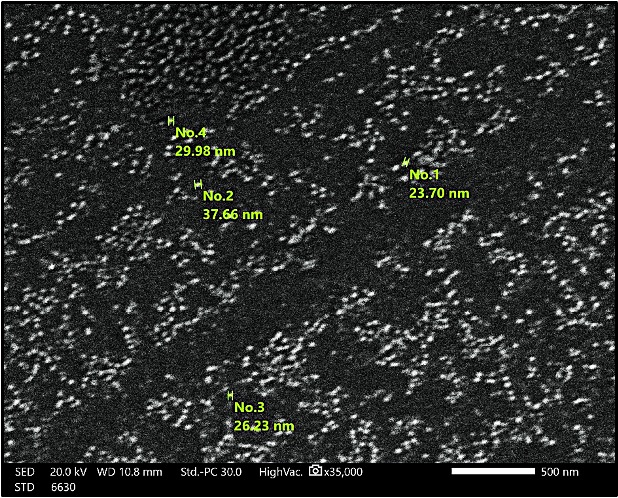

Supplement: Supplementary file 2 — Supplementary Material 2 [file 12903_2025_6299_MOESM2_ESM.jpg]

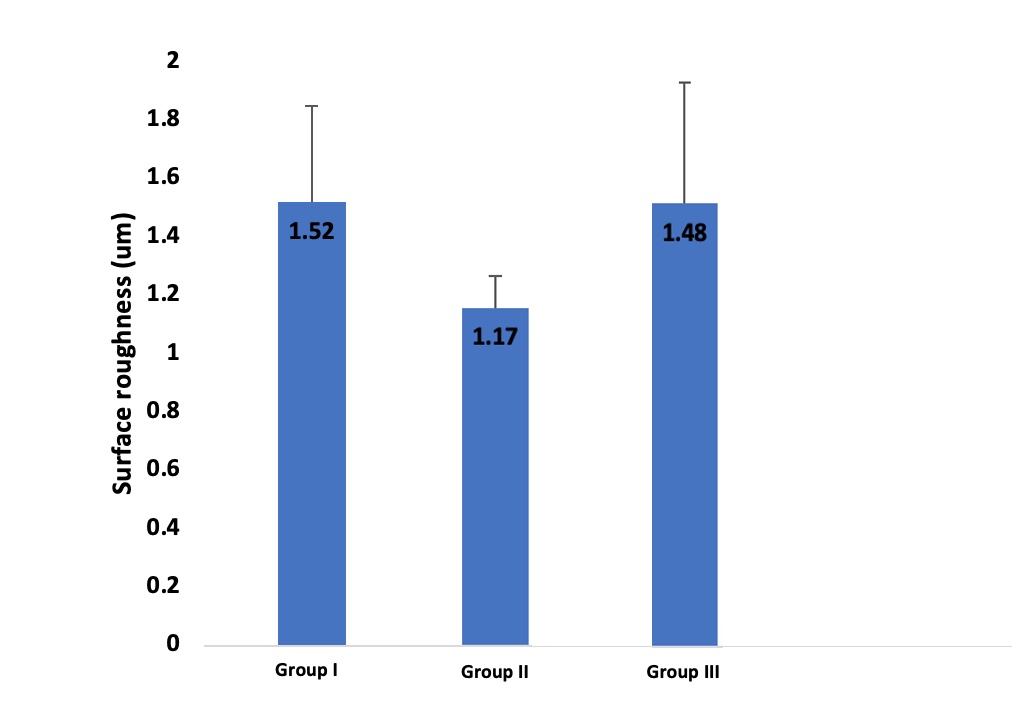

Supplement: Supplementary file 3 — Supplementary Material 3 [file 12903_2025_6299_MOESM3_ESM.jpg]

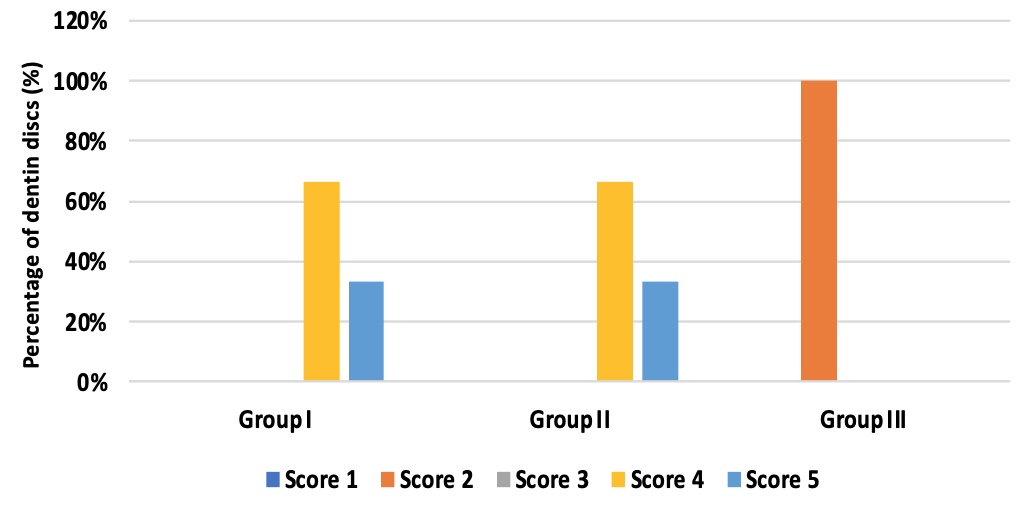

Supplement: Supplementary file 4 — Supplementary Material 4 [file 12903_2025_6299_MOESM4_ESM.jpg]

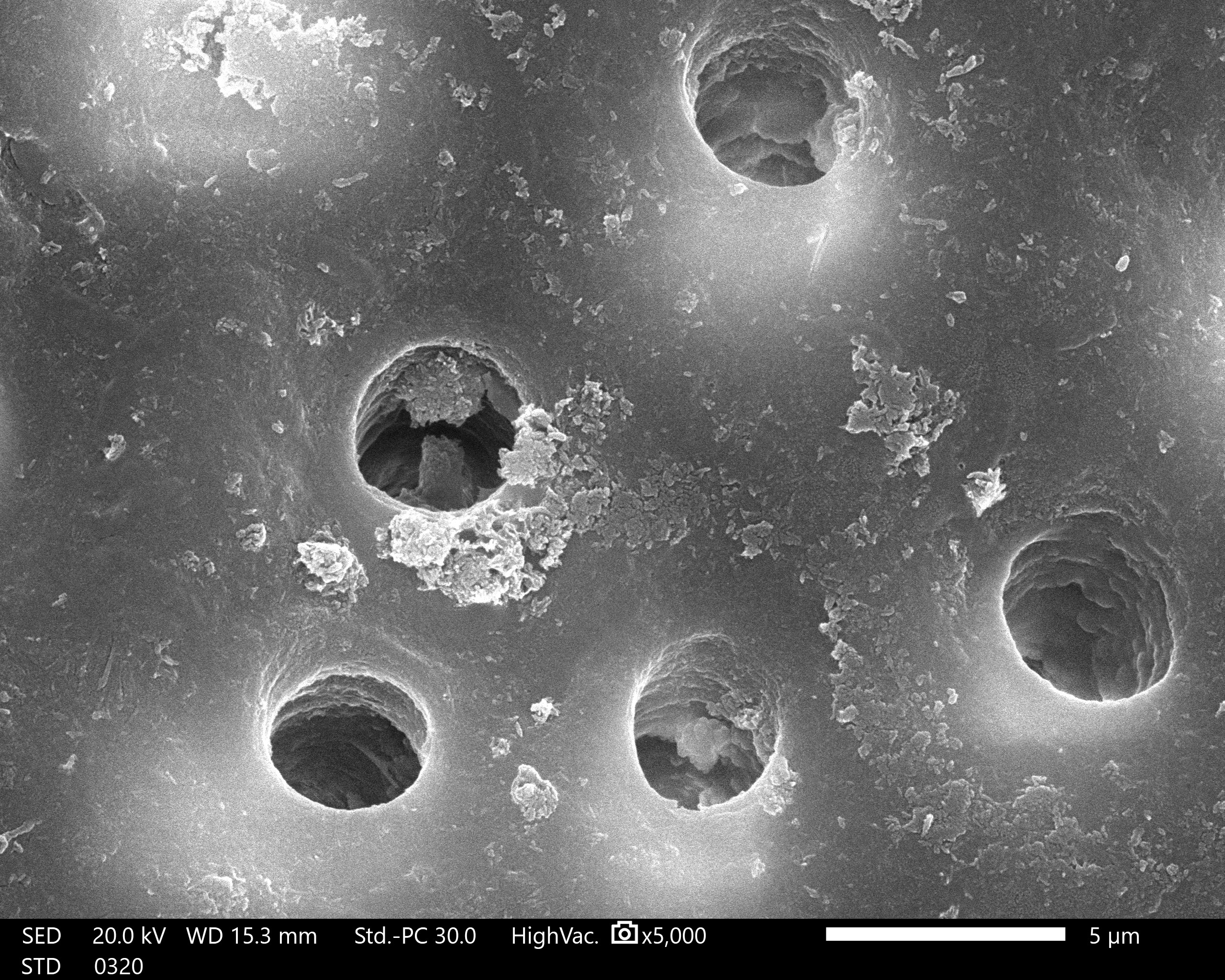

Supplement: Supplementary file 5 — Supplementary Material 5 [file 12903_2025_6299_MOESM5_ESM.jpg]

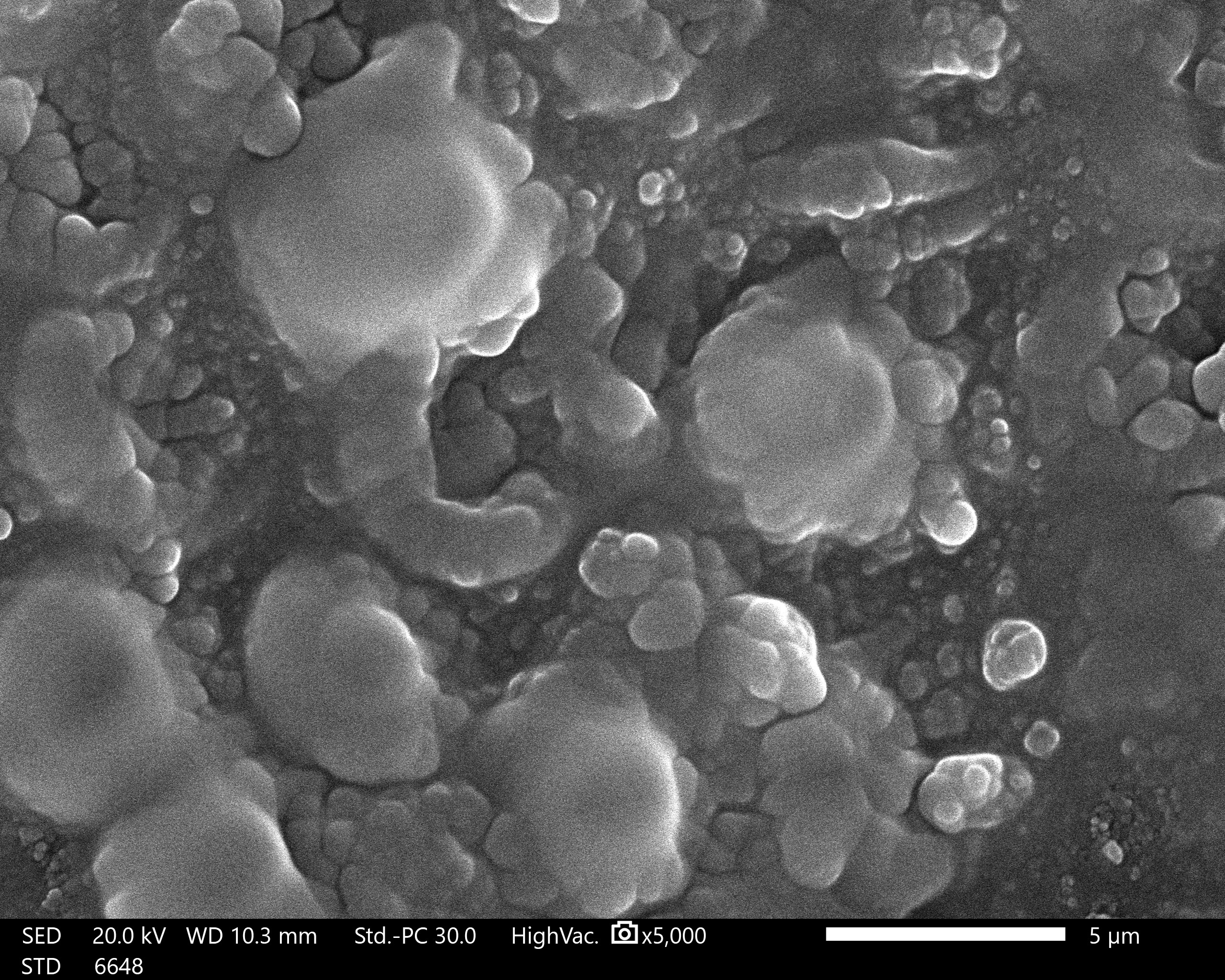

Supplement: Supplementary file 6 — Supplementary Material 6 [file 12903_2025_6299_MOESM6_ESM.jpg]
